# Study protocol: Phase I/II trial of induced HLA-G^+^ regulatory T cells in patients undergoing allogeneic hematopoietic cell transplantation from an HLA-matched sibling donor

**DOI:** 10.3389/fmed.2023.1166871

**Published:** 2023-05-19

**Authors:** Memnon Lysandrou, Dionysia Kefala, Panayiota Christofi, Nikolaos Savvopoulos, Penelope Georgia Papayanni, Rodanthy Theodorellou, Eleftheria Sagiadinou, Vassiliki Zacharioudaki, Maria Moukouli, Dimitrios Tsokanas, Georgios Karavalakis, Maria Liga, Konstantinos Stavrinos, Anastasia Papadopoulou, Evangelia Yannaki, Alexandros Spyridonidis

**Affiliations:** ^1^Bone Marrow Transplantation Unit and Institute of Cell Therapy, University of Patras, Rio, Greece; ^2^Gene and Cell Therapy Center, Hematopoietic Cell Transplantation Unit, Hematology Department, “George Papanikolaou” Hospital, Thessaloniki, Greece; ^3^Pharmassist Ltd., Athens, Greece

**Keywords:** allogeneic hematopoietic cell transplantation, graft versus host disease, adoptive cellular immunotherapy, regulatory T cells, HLA-G, hypomethylating agents, advanced therapeutic medicinal products (ATMPs)

## Abstract

Regulatory T-cell (Treg) immunotherapy has emerged as a promising and highly effective strategy to combat graft-versus-host disease (GvHD) after allogeneic hematopoietic cell transplantation (allo-HCT). Both naturally occurring Treg and induced Treg populations have been successfully evaluated in trials illustrating the feasibility, safety, and efficacy required for clinical translation. Using a non-mobilized leukapheresis, we have developed a good manufacturing practice (GMP)-compatible induced Treg product, termed iG-Tregs, that is enriched in cells expressing the potent immunosuppressive human leucocyte antigen-G molecule (HLA-G^+^). To assess the safety and the maximum tolerable dose (MTD) of iG-Tregs, we conduct a phase I–II, two-center, interventional, dose escalation (3 + 3 design), open-label study in adult patients undergoing allo-HCT from an HLA-matched sibling donor, which serves also as the donor for iG-Treg manufacturing. Herein, we present the clinical protocol with a detailed description of the study rationale and design as well as thoroughly explain every step from patient screening, product manufacturing, infusion, and participant follow-up to data collection, management, and analysis (registered EUDRACT-2021-006367-26).

## Background and rationale

Allogeneic hematopoietic stem cell transplantation (allo-HCT) has become a standard treatment for patients with marrow failure syndromes and hematologic malignancies such as acute leukemia. More than 20.000 allo-HCTs are performed every year in Europe with the numbers increasing (1). During allo-HCT, the patient is prepared with a conditioning regimen (e.g., chemotherapy and radiotherapy) to eliminate existing hematopoiesis and immune system followed by the administration of hematopoietic stem cells harvested from the donor. The graft will in turn engraft, proliferate, and finally reconstitute hematopoiesis and lymphopoiesis in the recipient (2). The therapeutic potential of this high-risk intervention relies on the ability of the engrafting immune system to mount a highly effective, alloreactive immune response against leukemia, termed the graft-versus-leukemia effect. However, this treatment is often accompanied by the occurrence of graft-versus-host disease (GvHD), a common life-threatening complication in which donor T cells attack the host’s normal tissues, which can occur early (acute, aGvHD) or later (chronic, cGvHD) after allo-HCT. GvHD can occur despite aggressive prophylaxis even when the donor is a matched HLA-identical sibling (3). Despite ground-breaking discoveries in GvHD pathobiology and advances in our understanding of the underlying mechanisms of this disease, conventional immunosuppressive pharmacotherapy remains the mainstay of GvHD prophylaxis and treatment. In particular, GvHD prophylaxis relies on the administration of calcineurin inhibitors, anti-T-lymphocyte agents, methotrexate, mycophenolate mofetil, or more recently post-transplantation cyclophosphamide. Acute and chronic GvHD treatment depends mostly on corticosteroids. Only during the past few years have other drugs gained approval as second-line treatment, such as Janus kinase 1/2 (JAK1/2), Bruton’s tyrosine kinase (BTK), or Rho-associated coiled-coil-containing protein kinase-2 (ROCK2) inhibitors, and real-life data for their efficacy need to be collected. GvHD and its currently available immunosuppressive treatment still pose the principal cause of post-transplant impairment of quality of life, morbidity (35–50%), and mortality (20–30%) after allo-HSCT, and in particular, patients with refractory GvHD have a dismal prognosis (4). Subsequently, the necessity for the development of novel preventive and therapeutic strategies is warranted.

A very promising alternative strategy against GvHD is immunotherapy using the adoptive transfer of T cells with regulatory properties (Tregs) as a living drug aiming to avoid prolonged pharmacological immunosuppression. There is convincing evidence in pre-clinical models and promising data from early-phase clinical trials that immunotherapy with naturally occurring Tregs (nTregs) is feasible, safe, and can suppress exuberant immune activation (5–9). However, their low numbers in the periphery and the lack of specific cell surface markers for efficient purification challenge the clinical translation of nTregs. We and other researchers attempt to overcome these hurdles by developing protocols for the *ex vivo* generation of stable induced Treg (iTreg) products of a defined phenotype that can be easily manufactured for clinical purposes. Some of these products have proceeded to phase I/II clinical studies showing the feasibility and safety of this approach with encouraging results (10, 11). Our approach aspired to mimic the physiological mechanism of the successful immune tolerance transpiring during pregnancy, where the human leukocyte antigen-G (HLA-G), a well-known immunoregulatory molecule, is expressed in the placenta, thereby protecting the “semi-allogeneic” fetus from maternal immune rejection (12). As the HLA-G gene is epigenetically repressed after prenatal life and the methylation status of the HLA-G promoter regulates its transcriptional activity, we showed in small-scale *in vitro* experiments that exposure of human peripheral T cells to hypomethylating agents (azacitidine or decitabine) induces a *de novo* and stable expression of HLA-G, in turn, converting them to Tregs with *in vitro* immunosuppressive functions (13, 14). Subsequently, we developed and validated the manufacturing process of an HLA-G^+^ regulatory T-cell-enriched product, termed iG-Tregs, that exerts its suppressive function through the HLA-G using a clinical scale and good manufacturing practice (GMP)-grade protocol (15). iG-Tregs can be consistently and robustly produced and display suppressive properties with a favorable safety profile both *in vitro* and *in vivo* (manuscript in preparation). Herein, we present the protocol of the ongoing phase I/II clinical study for the evaluation of iG-Tregs against GvHD in the context of HLA-matched sibling donor allo-HCT.

## Study setting

This is a phase I–II, two-center, interventional, dose escalation, open-label study of iG-Tregs as GvHD prophylaxis in adult patients undergoing HLA-matched sibling donor HCT (Group A). The study includes a dose escalation phase I cohort to define the maximum tolerable dose (MTD) and an extension phase II cohort at the selected MTD. The primary objective is to assess the safety of *ex vivo* generated iG-Tregs in adult patients undergoing HLA-matched sibling donor HCT and the secondary objective is to assess the clinical efficacy in preventing GVHD. Patient enrollment will also be enabled for an ancillary study for the clinical evaluation of iG-Tregs in the treatment of patients with cGvHD refractory to at least two lines of treatment (Group B; Supplementary Data).

The clinical study will be conducted at the bone marrow transplantation (BMT) unit of the University General Hospital of Patras (UGHP), Rio, Greece, and HCT Unit (HCTU), “George Papanicolaou” Hospital, Thessaloniki, Greece, which are both equipped with GMP facilities and allo-HCTs are routinely performed according to the Joint Accreditation Committee ISCT-Europe & EBMT (JACIE) standards (16). The current protocol has received approval from competent authorities (National Organization for Medicines: IS 129-22, National Ethics Committee: 181/22) and is registered in the European Union Drug Regulating Authorities Clinical Trials (EUDRACT) database (2021-006367-26).

## Study population

**Figure 1:**
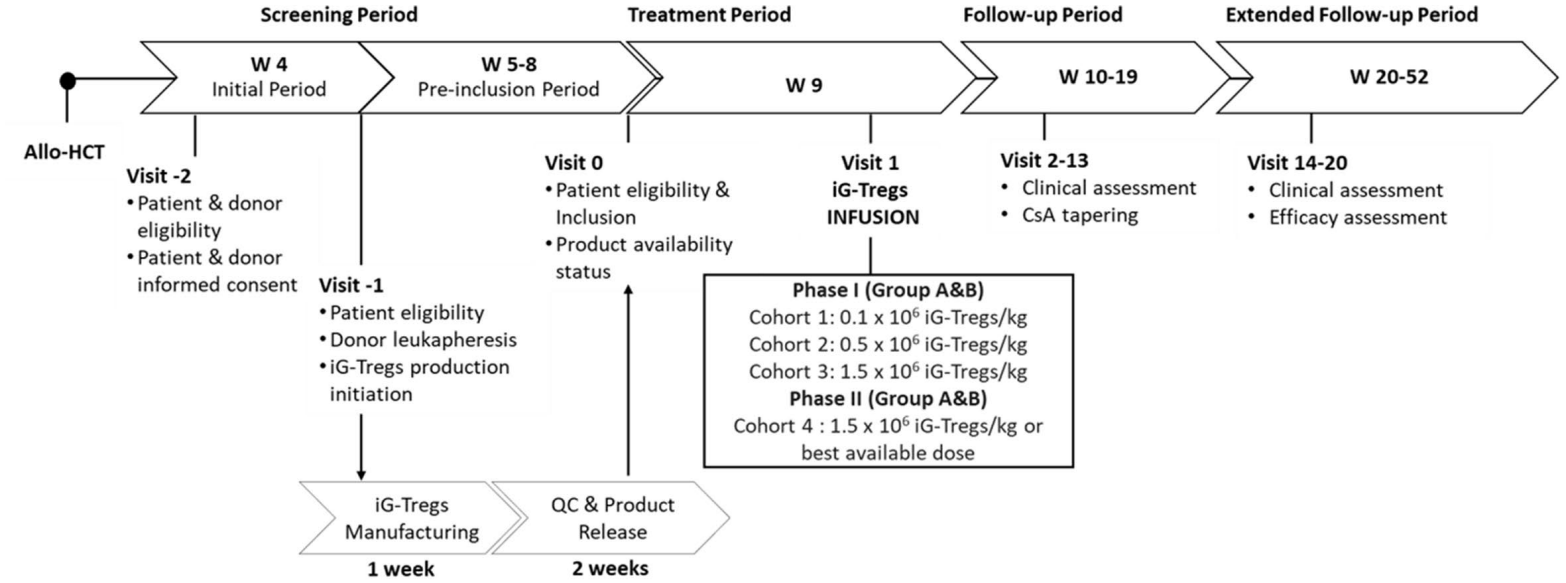
Participant timeline of the iG-Tregs phase I/II clinical trial (Group A). The day of the HLA-matched sibling allo-HCT defines the beginning of the study timeline. The screening period starts 4 weeks (W) following the transplant where the eligibility of the patient and donor is assessed, and informed consent is given. At W5, the sibling donor undergoes leukapheresis, and iG-Tregs production is initiated, which requires 1 week for manufacturing and 2 weeks for quality control (QC) and product release. At W5, the patient is re-evaluated for eligibility, and if the product is released and available, the patient is included. Upon inclusion, iG-Tregs infusion takes place within 1 week (W9) at a dose according to the current recruiting cohort. During the subsequent follow-up period (W10-19), the patient is continually assessed, and cyclosporine (CsA) tapering is allowed from W13 onwards. Finally, for the extended follow-up period (W20-52) patient clinical evaluation continues and the efficacy of iG-Tregs is assessed. Allo-HCT, allogeneic hematopoietic cell transplantation; Group A refers to the patients recruited for the main study for GvHD prophylaxis; Group B refers to the patients recruited for the ancillary study for chronic GvHD treatment.

Recruitment of patients focuses on adults (>16 years of age) who undergo an allo-HCT from an HLA-matched sibling donor. The sibling donor will provide the starting material for iG-Treg manufacturing. All patients will be evaluated for eligibility after allo-HCT, along with the availability and eligibility of the sibling donor for leukapheresis at day (d) 30 after allo-HCT. The sample size is determined based on the “3 + 3” design (17). Briefly, patients will receive the iG-Tregs in cohorts of three (Cohort 1: 0.1 × 10^6^ iG-Tregs/kg; Cohort 2: 0.5 × 10^6^ iG-Tregs/kg; and Cohort 3: 1.5 × 10^6^ iG-Tregs/kg), in a dose escalation fashion if no dose-limiting toxicity (DLT) is documented. The occurrence of DLT in one patient leads to the extension of the cohort to a total of six patients, whereas the presence of DLT in more than one patient of a given cohort denotes that the MTD has been surpassed. During phase ΙΙ, the enrollment of an additional eight patients and infusion of the MTD or best available dose (Cohort 4) will take place to collect further information concerning the safety profile (Figure 1). Collectively, a maximum of 26 patients could be enrolled in this study.

### Patient inclusion criteria

1. Previous HLA-matched sibling allo-HCT at least 30 days before.
2. The following criteria must be fulfilled at the initial assessment and on the day of the iG-Treg infusion:

- Performance status: Karnofsky ≥80%.
- Adequate hematopoiesis and organ function.
- Negative pregnancy test (female patients).

3. Ability to understand and willingness to sign an informed consent form.

Additional inclusion criteria for the Group B patients enrolled in the ancillary study can be found in the Supplementary Data.

### Patient exclusion criteria

1. History of a GVHD grade ≥ II [according to the MAGIC criteria (18)] or administration of any first-line systematic treatment against aGvHD.
2. Patients with evidence of residual disease during the final assessment.
3. Active serious infections not responding to treatment.
4. HBV-, HCV-, or HIV-positive patients.
5. Administration of any investigation drug/product ≤28 days prior to iG-Treg infusion.

### Donor inclusion criteria

1. The HLA-matched sibling donor who donated the graft for the allo-HCT of the corresponding patient will be assessed and fulfill the requirements according to institution procedures and JACIE standards to undergo leukapheresis (donor lymphocytes) without prior granulocyte colony-stimulating factor (G-CSF) infusion.
2. Age between 16 and 75 years.
3. Body weight > 40 kg and in good general health.
4. The donor must be able to understand and be willing to sign an informed consent form.
5. Negative pregnancy test (female donors).

## Study timeline

### Allo-HCT

The day of graft infusion is defined as d0. Succeeding the allo-HCT, the patient serially follows carefully planned visits through the screening, follow-up, and extended follow-up periods (Figure 1).

### Screening period

#### Visit-2 (week 4 post-allo-HCT)—Patient and donor eligibility

During this visit, the patient and the sibling donor are checked for inclusion eligibility. The consent form is signed and collected.

#### Visit-1 (week 5 post-allo-HCT)—donor leukapheresis

The patient is screened to meet all eligibility criteria, and the sibling donor undergoes leukapheresis for the initiation of the iG-Tregs manufacturing. The donor undergoes steady-state leukapheresis according to institutional guidelines for less than 1 h yielding at least 60 mL of the leukapheresis product.

#### iG-Tregs manufacturing period (weeks 5–8 post-allo-HCT)

The leukapheresis product will be transferred to the GMP Unit within 24 h in order to generate the final iG-Treg product, according to a GMP-compliant protocol (15). Briefly, T cells are enriched from peripheral blood mononuclear cells, then activated for 3 days using an anti-CD3/CD28 activation agent (GMP TransAct, Miltenyi), and finally undergo hypomethylation treatment with decitabine (Dacogen, Janssen) in the presence of interleukin-2 (Miltenyi) for an additional 3–4 days. The final product is thoroughly washed four times with 10% human serum albumin in Hank’s balanced salt solution prior to the final formulation (weeks 5–6). The final cell product will first undergo quality control (QC) testing to assess cell numbers, viability, and HLA-G^+^ content, and the transplant center will be informed of product manufacturing completion. Each iG-Tregs product comprises a unique batch of cells intended for a specific patient. The appropriate cell dose depending on the cohort is cryopreserved. Finally, the product goes through rigorous QC testing for the final release (weeks 6–8; Table 1).

**Table 1:** Quality control testing of the final iG-Tregs product.

Quality control test	Method	Criteria	Purpose
Sterility	Cultures for aerobic and anaerobic bacteria	Negative at week 7	Final release	Cultures for fungi	Negative at week 8
	Mycoplasm	Biochemical assay	Negative	Final release
Endotoxin levels	LAL test	<5.0 EU/kg	Final release
Immunophenotype	Flow Cytometry	CD3 + ≥ 80%, alive HLA-G+ cells ≥10%	Product manufacturing
Cell number	Cell counting using hemocytometer	Cell Number ≥ 0,1*10^6^ /kg	Product manufacturing
Viability	Trypan Blue staining	Viable cells ≥50%	Product manufacturing

#### Visit 0 (week 8)—patient inclusion

The patient is reassessed to meet all eligibility criteria, and final product availability is confirmed. If the criteria are met, the patient is scheduled to receive the infusion of iG-Tregs within a week.

### Treatment period

#### Visit 1 (week 9 post-allo-HCT)—iG-Tregs infusion

Thawing and intravenous infusion of iG-Tregs are performed bedside by an experienced physician. Prior to infusion, the patient will receive antihistaminic prophylaxis (dimetindene). Patients are closely monitored (pulse oximetry and vital signs) for 1 h post-infusion. If any toxicity and/or adverse event (AE, graded according to CTCAEv.4) occurs, this will be recorded and managed appropriately. Participants will be instructed to avoid steroids up to 1 week after the iG-Tregs infusion.

### Follow-up period

#### Visits 2–13 (weeks 10–19 post-allo-HCT)

At each weekly visit, the patient is evaluated for any AE and the occurrence of aGvHD. It is emphasized that there are no drug restrictions regarding supportive care, or other treatment to prevent disease relapse while steroids should be avoided for at least up to 1 week following iG-Tregs administration. From week 13 and onward, should the patient’s status allow, a cyclosporine (CsA) tapering of a 25% weekly dose reduction can be applied until complete cessation.

### Extended follow-up period

#### Visits 14–20 (weeks 20–52 post-allo-HCT)

The patient will be evaluated at predefined time intervals according to the investigational sites’ clinical practice protocol and the observed clinical course.

## Outcomes

The current clinical trial aims to define the safety, tolerability, and MTD of the iG-Tregs (within a timeframe of 90 days following the infusion) as well as collect data regarding its clinical efficacy (within a time frame of 52 weeks following allo-HCT). The primary outcomes of this study include the (1) incidence of infusion toxicity (within 1 h after the infusion), (2) additional toxicities that may occur related to iG-Tregs infusion (e.g., the occurrence of exacerbation of GvHD, infections, and disease relapse), and (3) AEs occurring during the first 3 weeks following iG-Treg infusion, which will be accounted for the assessment of the safety profile and tolerability of each dose during the dose escalation phase.

The secondary outcomes of this study include the (1) incidence and severity of GvHD, (2) day of CsA cessation, and (3) treatment failure (includes the diagnosis of GvHD, inability to cease CsA administration until d + 150 following allo-HCT, and disease relapse).

Additional secondary outcomes for patients (Group B) enrolled in the ancillary study can be found in the Supplementary Data.

## Interruption and early termination of the study

The sponsor reserves the right to suspend enrollment or terminate the study at any time as defined in the clinical study agreement for reasons including, but not limited to, insufficient data collection, low participant enrollment rate, achievement of full enrollment, conditions imposed by the regulatory authorities, non-compliance with the clinical trial protocol, or medical reasons.

## Statistical methods

Given the small numbers, events will be summarized using descriptive statistics, such as frequencies and proportions. GVHD and relapse rates will be estimated as cumulative incidence curves, death in remission as a competing risk for relapse, and death without GVHD as a competing risk for GVHD. Estimates of overall survival (OS) and relapse-free survival (RFS) will be obtained by the method of Kaplan–Meier. Differences between subgroups will be compared using the Fisher exact test for categorical data and the Mann–Whitney U-test for continuous data. Statistical significance was based on *p* < 0.05.

## Data collection, management, and monitoring

All required clinical data of this trial will be collected on standardized patient follow-up forms. Confidentiality will be maintained in accordance with current clinical research principles and the General Data Protection Regulation (GDPR), and participants’ personal information will be in a pseudonymous format. The study will be monitored by the clinical trial quality assurance company (Contract Research Organization, CRO). Data will be examined for protocol compliance and accuracy against source documents.

## Research studies

Additional research studies will be conducted in conjunction with the clinical assessment of enrolled patients. Specifically, extensive patient immunophenotyping using flow cytometry, and iG-Tregs evaluation for effector cytokine production, alloreactivity *in vitro* and *in vivo,* and immune suppressive function both *in vitro* and *in vivo* will be performed. Moreover, released products will undergo T-cell receptor (TCR) repertoire next-generation sequencing (NGS)-based analysis for further characterization and *in vivo* tracking. Finally, serum cytokine levels will be monitored at multiple time points following product infusion.

## Discussion

The present protocol aims to assess the clinical translation of a novel iTreg product targeting GvHD post-allo-HCT. Several groups have sought to prevent potentially lethal GvHD by employing adoptive immunotherapies either based on nTregs or iTregs (6–8, 10, 11). Despite the differences in product manufacturing and trial design, previous studies have paved the way for such clinical applications with promising results (19).

Throughout these protocols variability in donor selection, which have been either matched siblings (6, 11), haploidentical (8), or mismatched donors (10) is apparent. Moreover, for nTregs, these approaches have relied mostly on fresh isolation and administration (6, 8) while iTreg products have both been infused as fresh (11) or cryopreserved (10). As far as manufacturing is concerned, fresh isolation of nTregs requires specialized equipment for immunomagnetic isolation and/or flow sorting, whereas iTregs demand laborious time-consuming manufacturing with extensive QC for release. Treg products are commonly diverse in content with the dominant regulatory population varying per patient batch. For example, initial approaches with CD4^+^CD25^+^ selection were accompanied by a high proportion of activated conventional T cells, which were finally co-infused along with the intended nTreg population (8). Overall, even though these trials have recruited low numbers of patients, a phase III trial is currently ongoing, exhibiting encouraging results (7). In our study, the iG-Tregs product is manufactured consistently through a short, simple, and robust protocol (15). Notably, iG-Tregs contain a distinct regulatory population characterized by the surface expression of HLA-G, which enables easy *in vivo* tracking. Subsequently, the presence of HLA-G apart from mediating the suppressive function can serve as a selection marker for in-depth analysis to unravel novel modes of action. This, in turn, will shed light on which mechanism holds clinical relevance and ultimately lead to process refinement and targeted cell engineering.

Up to date, most completed and ongoing trials employing Treg immunotherapies for GvHD in the setting of allo-HCT have focused on prevention. These strategies depicted the production feasibility and safety of these cell therapies by determining the MTD at phase I studies. Common ground for these trials lies upon the infusion of the final product during the peri-transplantation period (day −4 to day 0 of allo-HCT), with the aim of promoting proper immune reconstitution. In our study, product infusion takes place at week 9 post-allo-HCT and not during the peri-transplantation period. Following MTD determination, we shall assess clinical efficacy through the ability to taper and finally cease CsA administration at an earlier time point in contrast to others, where GvHD occurrence was the main parameter. Moreover, previous work has described the effect of immunosuppressive prophylaxis—targeting calcineurin and the mammalian target of the rapamycin (mTOR) pathway—on the function of nTregs (20, 21). Hence, we cannot exclude the possibility of CsA interfering with the function of iG-Tregs, something which we are currently evaluating *in vitro* and *in vivo*. Finally, in an ancillary study, we will evaluate the safety and applicability of iG-Tregs immunotherapy as a third-line treatment for cGvHD.

Collectively, the ongoing phase I/II clinical trial of iG-Tregs constitutes an innovative approach of iTreg immunotherapy in patients undergoing allo-HCT from an HLA-matched sibling donor.

## Dissemination policy

The trial has been registered in the EUDRACT registry prior to the inclusion of the first participant to meet the regulatory requirements (EUDRACT-2021-006367-26). After the conclusion and final analysis of the trial data, results will be submitted to a peer-reviewed medical scientific journal.

## Ethics statement

The clinical trial is conducted in accordance with the ethical principles for medical research referred to in the Declaration of Helsinki by the World Medical Association and all applicable Greek and European laws and regulations regarding clinical drug trials, including the Principles of Good Clinical Practice (GCPs). Approval was obtained by jurisdictional ethics committees (National Ethics Committee and Institutional Review Boards of UGHP, University of Patras, and George Papanikolaou Hospital) and the Greek National Organization for Medicines (IS 129-22). The Investigator will submit and, where necessary, obtain approval from the aforementioned authorities for all material amendments to the original approved documents.

## Author contributions

AS: principal investigator. EY and AS: primary investigators of centers. MLy, RT, MM, KS, EY, and AS: clinical protocol design. MLy, DK, PC, NS, PP, and AP: manufacturing processes. ES, VZ, DT, GK, and MLi: clinical study procedures. MLy, DK, PC, NS, PP, RT, ES, VZ, MM, DT, GK, MLi, KS, AP, EY, and AS: project planning. MLy, DK, and AS: manuscript preparation—initial draft. All authors contributed to the article and approved the submitted version.

## Funding

This research has been co-financed by the European Union and Greek national funds through the Operational Program Competitiveness, Entrepreneurship, and Innovation, under the call “RESEARCH—CREATE—INNOVATE (project code: T2EDK–02437).”

## Conflict of interest

RT, MM, and KS were employed by Pharmassist Ltd.

The remaining authors declare that the research was conducted in the absence of any commercial or financial relationships that could be construed as a potential conflict of interest.

## Publisher’s note

All claims expressed in this article are solely those of the authors and do not necessarily represent those of their affiliated organizations, or those of the publisher, the editors and the reviewers. Any product that may be evaluated in this article, or claim that may be made by its manufacturer, is not guaranteed or endorsed by the publisher.
