# Supplementary material for: Study protocol: Phase I/II trial of induced HLA-G+ regulatory T cells in patients undergoing allogeneic hematopoietic cell transplantation from an HLA-matched sibling donor
Source: Front Med (Lausanne). 2023 May 19;10:1166871. doi: 10.3389/fmed.2023.1166871 (PMC10237041; doi:10.3389/fmed.2023.1166871)
Supplement: Supplementary file 1 [file Data_Sheet_1.docx]

Supplementary Material

Study Protocol: Phase Ι/II trial of induced HLA-G^+^ regulatory T cells (iG-Tregs) in patients undergoing allogeneic hematopoietic cell transplantation from an HLA-matched sibling donor

Memnon Lysandrou^1*^, Dionysia Kefala^1*^, Panayiota Christofi^1,2^, Nikolaos Savvopoulos^1^, Penelope Georgia Papayanni^2^, Rodanthy Theodorellou^3^, Eleftheria Sagiadinou^1^, Vassiliki Zacharioudaki^1^, Maria Moukouli^3^, Dimitrios Tsokanas^1^, Georgios Karavalakis^2^, Maria Liga^1^, Konstantinos Stavrinos^3^ Anastasia Papadopoulou^2^, Evangelia Yannaki^2#^, Alexandros Spyridonidis^1#^

^1^ Bone Marrow Transplantation Unit & Institute of Cell Therapy, University of Patras, Rio, Greece

^2^ Gene and Cell Therapy Center, Hematopoietic Cell Transplantation Unit, Hematology Department, “George Papanikolaou” Hospital, Thessaloniki, Greece

^3^ Pharmassist Ltd, Athens, Greece

* Co-first authorship

# Co-senior authorship

**Correspondence:**

Prof. Alexandros Spyridonidis, MD, PhD, Bone Marrow Transplantation Unit and Institute of Cell Therapy, University of Patras, 26504, Rio, Greece (email: spyridonidis@upatras.gr)

# Supplementary Data

**Ancillary study for clinical evaluation of iG-Tregs in the treatment of cGvHD refractory to second-line treatment**

### *Study Population*

During this ancillary study, cases of patients diagnosed with moderate or severe cGvHD after allo-HCT, who will be treated with steroids and at least one other second-line (2L) therapy (Group B), will be evaluated for inclusion in the study, on the day of initiation of treatment for cGvHD. On the same day, the sibling donor will be evaluated for leukapheresis.

*Additional Inclusion Criteria:*

The patient inclusion criteria (Group B) are as follows:

1. Diagnosis of moderate or severe cGvHD according to the 2014 updated National Institutes of Health (NIH) criteria to be treated with steroids and at least one other 2L therapy,
2. Diagnosis of cGvHD resistant to second-line treatment defined as the progression of cGvHD or stable disease after prednisone ≥0.5 mg/kg/day (or equivalent dose of alternative glucocorticoids) given together with 2L therapy for at least 2 weeks OR prednisone ≥0.5 mg/kg/day for at least 4 weeks given as monotherapy AND 2L therapy for at least 2 weeks administered as monotherapy
3. Fixed treatment dose of 2L for 2 weeks before enrollment. 2L therapy can be continued and prednisone should not exceed 0.5 mg/kg/day.
4. 2L therapies include but are not limited to ruxolutinib ≥ 10mg/kg daily, mycophenolate-mofetil ≥ 1 g/d, ibrutinib, mTOR inhibitors, sirolimus and CsA at levels >200ng/ml. Continuous prophylactic calcineurin inhibitor counted as GvHD treatment only if the dose was increased >50% on the previous prophylactic dose. T cell-depleting strategies (e.g ATG, alemtuzumab) are excluded.

### *Ancillary Study Timeline*

Screening of patients for this ancillary study is performed on the day of treatment initiation for moderate or severe cGvHD according to the NIH update 2014 criteria. At the same time, the sibling donor is also evaluated for leukapheresis. Within 7 days, the patient will be re-evaluated and if he/she has either disease progression on first-line therapy or stable disease on second-line therapy, the leukapheresis procedure will be performed on the sibling donor. Infusion of the iG-Tregs product will be performed 7 +/-3 days after leukapheresis if patients meet all eligibility criteria. Follow-up of patients post iG-Tregs infusion lasts 52 weeks and during this period the patient is closely monitored for AE occurrence and GvHD status. The study ends at the completion of the 52-week follow-up after iG-Tregs infusion.

### *Additional Outcomes*

Beyond the primary outcomes of the main study on safety, tolerability and MTD, additional secondary outcomes will be assessed concerning the efficacy of iG-Tregs in the treatment of cGvHD refractory to second-line treatment (Time Frame: 52 weeks post infusion). Specifically, (1) participants' cGvHD response at 1, 2, 4, 6, 8, 12 and 26 weeks after iG Treg infusion with overall response defined as either complete, partial response or stable disease (complete response is defined as the resolution of all reversible cGvHD-related events in a given organ, partial response is defined as at least 25% absolute or 50% relative change -whichever is greater- when comparing baseline and end measurements in one cGvHD event without worsening in the other events, progressive cGvHD -includes initiation of steroids or any other GvHD therapy-, treatment failure includes progressive cGvHD, stable disease, steroid dependence and disease relapse) and (2) Disease Free Survival (DFS), Transplant Related Mortality (TRM) and Overall Survival (OS) will be accounted for.
